# Setting Mechanical Insufflation-Exsufflation (MI-E) Pressures for Amyotrophic Lateral Sclerosis (ALS) Patients to Improve Atelectasis and Reduce Risk of Pneumothorax: A Case Report

**DOI:** 10.7759/cureus.25786

**Published:** 2022-06-09

**Authors:** Keiichi Funo, Yuri Negishi, Chika Akamine, Ryoko Takeuchi, Yoshihiro Uzawa

**Affiliations:** 1 Rehabilitation, Kameda Medical Center, Kamogawa, JPN; 2 Neurology, Kameda Medical Center, Kamogawa, JPN

**Keywords:** mechanical ventilation, neuromuscular disease, atelectasis, amyotrophic lateral sclerosis, mechanical insufflation-exsufflation, respiratory care, airway clearance

## Abstract

Mechanical insufflation-exsufflation (MI-E) has been used to supplement the ability to cough and expel pulmonary secretions in patients with neuromuscular disease who have a reduced ability to cough. The manufacturer's guidelines for MI-E recommend a setting of inspiratory pressure of +40 cmH_2_O and expiratory pressure of -40 cmH_2_O. However, patients with small stature and restricted ventilatory impairment are prone to pneumothorax, so the manufacturer's recommendations are not used as is, and should be adjusted for the physical and pulmonary characteristics of each patient. Here, we report a case in which MI-E was used for an amyotrophic lateral sclerosis (ALS) patient with short height, low BMI, and restricted lung capacity at inspiratory and expiratory pressures lower than the manufacturer's recommendations. In adjusting MI-E pressure, physical observations such as chest auscultation, visual chest dilation, and observation of secretion movement toward the tracheal tube were performed to avoid unnecessary pressure. As a result, the pressure level set was lower than the manufacturer's recommendation (25 cmH_2_O) but sufficient to improve atelectasis and no pneumothorax occurred. The method we practiced in this study is feasible in any clinical setting. We also believe that MI-E, when performed in conjunction with treatment response observation, can be expected to improve at lower pressures than generally recommended, thereby reducing the risk of lung injury and providing safer treatment.

## Introduction

Amyotrophic lateral sclerosis (ALS) is a progressive neurodegenerative disease that affects upper and lower motor neurons, resulting in generalized movement disorders, respiratory muscle dysfunction, dysarthria, and dysphagia [1,2]. Pulmonary complications of pneumonia, atelectasis, and respiratory failure often result in morbidity and mortality due to impaired cough and the ability to clear pulmonary secretions [1,3]. Reducing such complications may improve dyspnea and extend patients’ lives. Mechanical insufflation-exsufflation (MI-E) devices have been used in palliative care for other neuromuscular disorders (NMD) patients to compensate for their decreased ability to cough and expectorate pulmonary secretions. MI-E is a device that applies positive pressure to the airways and lungs and then rapidly switches to negative pressure to promote expectoration by rapidly withdrawing air from the upper airways [4]. Evidence for the effectiveness of this non-pharmacologic therapy has resulted in published clinical practice guidelines for applying airway clearance therapy in NMD patients [5].

There are concerns about the risk of complications with the application of MI-E. These include abdominal distention, discomfort, gastroesophageal reflux, pneumothorax, and cardiovascular effects such as blood pressure changes and cardiac arrhythmias [6]. Loewen et al. reviewed a case series that gave insight into the pneumothorax complication with the use of MI-E. The report identified characteristics of patients who developed pneumothorax and had an average weight of 43 kg, BMI of 16 kg/m^2^, and vital capacity (VC) of approximately 1.0 L. This report implied that having reduced body weight, low BMI, and restricted lung capacity increased the risk for pneumothorax [7]. MI-E manufacturers’ guidelines recommended inspiratory pressure settings of +40 cmH_2_O and expiratory pressure -40 cmH_2_O [8,9]. These generic guidelines do not consider each patient's physical and pulmonary characteristics and should be used with caution. Systematic reviews of the literature have identified insufficient evidence for both important outcomes as well as complications with MI-E and NMD patients [10,11].

In this case report, we review our experience with an ALS patient with short stature, low BMI, and restricted lung capacity who needed MI-E to treat pneumonia and atelectasis. We used lower inspiratory and expiratory pressure settings than what the manufacturer recommended. We found the reduced pressure levels were sufficient to improve atelectasis and no pneumothorax occurred. We believe that by using this individualized approach, MI-E can still be effectively implemented for a low height and low BMI patients with reduced risk of barotrauma.

## Case presentation

Case introduction

We saw an 81-year-old woman who developed weakness in her left lower limb four years prior to admission, and she was diagnosed with ALS two years before admission, then edaravone and antioxidants were initiated. Six months before admission, an endoscopic gastrostomy was performed for nutritional management due to the progression of dysphagia. She also had endoscopic submucosal dissection (ESD) for early-stage gastric cancer four months prior to admission. There is no other notable history of surgery or medical history. Activities of daily living (ADL) were almost entirely assisted by family members, but her arms and fingers are mobile despite muscle weakness, so writing and smartphone communication have been possible. She was 149 cm tall, weighed 42.3 kg, and had a BMI of 19.1 kg/m^2^. A pulmonary function test measured one month before admission showed a vital capacity of 0.67 L and a %VC of 33.5%. Six months before admission she had started using MI-E (Cuff Assist E70, Philips Respironics) as her respiratory function had gradually declined. The device was performed via mask to assist expectoration airway secretion. MI-E was also performed by family members with training from a medical practitioner. Her recent admission to our hospital was due to worsening respiratory complications with a diagnosis of bilateral lower lobe pneumonia and atelectasis.

This case report was approved by the institutional review board (approval 22-002), and appropriate written informed consent was obtained for the patient.

Course of treatment

**Figure 1:**
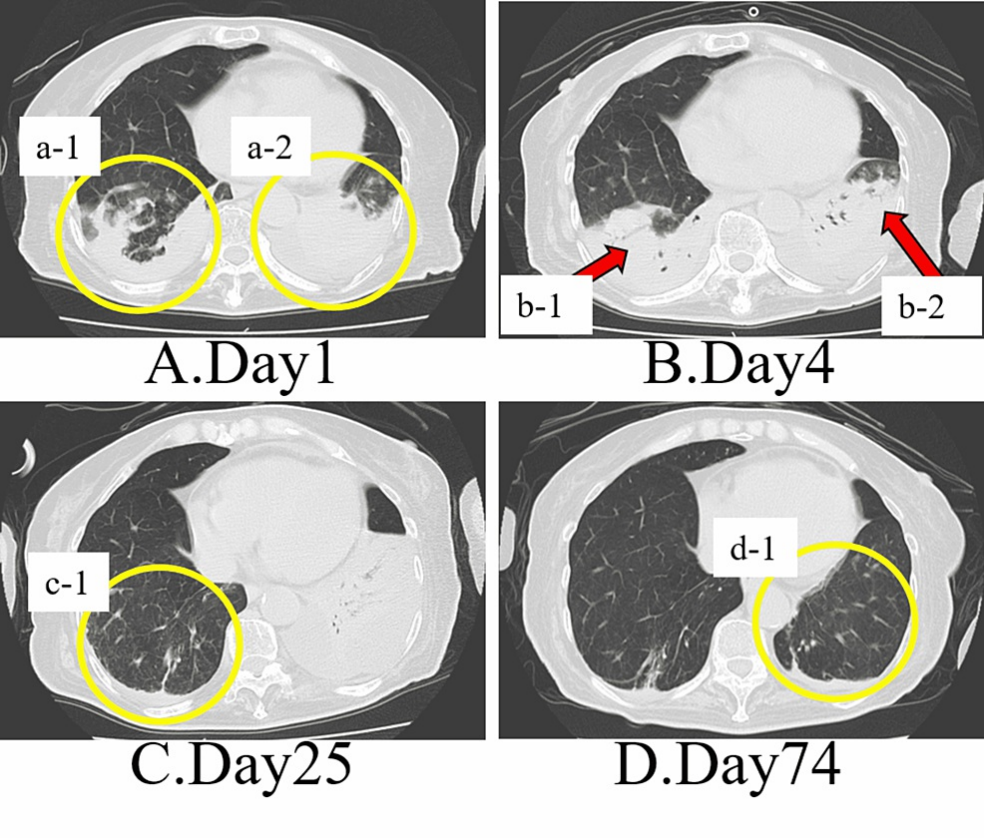
Chest CT on hospital admission day 1 (A), day 4 (B), day 25 (C), and day 74 (D). A. Bilateral pleural effusions, a small amount of pericardial effusion, and atelectasis in the bilateral lower lobes. Pneumonia is seen in the bilateral lower lobes (yellow circle a-1, a-2). B. Mildly increased left pleural effusion. Infiltrating shadows in the bilateral lower lobes are enlarged in extent and show exacerbation of pneumonia (red arrow b-1, b-2). C. Infiltrating shadow, atelectasis, and pleural effusion in the right lower lobe improved (yellow circle c-1). D. Improvement of infiltrating shadow and atelectasis in the left lower lobe (yellow circle d-1).

**Figure 2:**
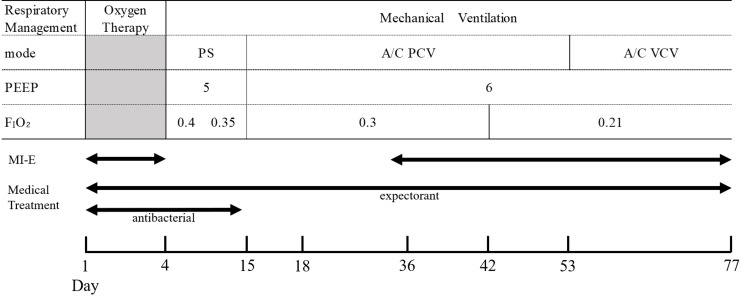
Progress chart PS: pressure support; A/C: assist/control; PCV: pressure-controlled ventilation; VCV: volume-controlled ventilation; PEEP: positive end-expiratory pressure; F_I_O_2_: inspiratory O_2_ fraction

On the first day of admission, pulmonary secretions were yellow and quite viscous. Laboratory studies revealed a C-reactive protein (CRP) level of 9.3 mg/ml and a white blood cell count (WBC) of 18000/μl. Computed tomography (CT) (Figure 1) showed atelectasis and pneumonia in both lower lobes, and bilateral pleural effusions predominantly on the left side. She was prescribed antimicrobials, nebulized expectorants, MI-E, and physiotherapy. On the fourth hospital day, her respiratory condition declined due to aspiration and greater difficulty in expectorating sputum; repeated CT showed worsening atelectasis in the left lower lobe (Figure 1), which prompted intubation and mechanical ventilation. On day 14, the sputum was white viscous, the CRP level was 0.9 mg/ml, and the white blood cell count was 7700/μl. On day 18, a tracheostomy was performed for the purpose of airway management. A tracheal cannula with a 7.5-mm inner diameter was inserted, and the patient continued on the mechanical ventilator. At this time, lung compliance was 30 ml/cmH_2_O. Figure 2 shows the progress of mechanical ventilator setting and medication.

MI-E protocol

After a tracheostomy was performed, MI-E was resumed. The pressure settings were adjusted based on those previously used as part of her initial physical assessment. This patient had used MI-E before admission to the hospital via mask, with inspiratory pressure +20 cmH_2_O and expiratory pressure -20 cmH_2_O. We increased previous pressure settings, to inspiratory pressure +25 cmH_2_O and expiratory pressure -25 cmH_2_O. Therapists confirmed lower lobe respiratory sounds by chest auscultation and adequate chest expansion by visible observation. Migration of pulmonary secretions toward the larger airways was noted. To confirm the movement of the secretions, therapists palpated the tracheal area to check whether rattling could be felt and auscultated for rhonchi. When such findings were found, therapists performed tracheal suctioning. From these observations, a pressure of ±25 cmH_2_O was finally determined. For the rest of the settings, the inspiratory and expiratory pause time was 2 seconds, and inspiratory and expiratory time (TI& TE) were also set at 2 seconds as standard fashion. The numbers of repetitions of the MI-E were 3-5 times in each session. The length of the therapy varied from 15 to 20 minutes depending on the state of sputum expectoration as well as the patient’s level of tolerance and fatigue. Rest time between sets was provided for suctioning secretions from tracheostomy and oropharynx. MI-E therapy was conducted 2-3 sessions a day. Pulse oximetric saturation (SpO_2_) and pulse rate were monitored during each MI-E procedure. Peak cough flow (PCF) which is the highest flow during exhalation, was also monitored. On the previously noted settings, PCF was 140 L/min. Due to bilateral but left dominant atelectasis, the patient was placed in the right lateral recumbent position during MI-E for the postural drainage effect. Additional chest CT imaging was performed three times during hospitalization to monitor the effectiveness of treatment.

Physical therapy course

Physical therapy was prescribed and begun on day 3. Physical and occupational therapists performed positional drainage and rib cage compression as chest physical therapy. Passive range of movement assistive exercises was used to prevent joint contractures. In addition, the patient was transferred to a sitting position at the edge of the bed and a wheelchair with assistance. During the day, the patient spent two to three hours seated in a wheelchair. On hospital day 36, a glottis closure was performed to prevent aspiration and to improve oral intake. A tracheal cannula was changed to an 8.0-mm inner diameter. Nebulized expectorants, MI-E, and respiratory physiotherapy were continued. As a result of the continued therapeutic interventions, on hospital day 74, the CT showed improvement in atelectasis (Figure 1), and lung compliance had increased to 40 ml/cmH_2_O. The patient’s ability to oxygenate had improved which allowed the fraction of inspired oxygen (F_I_O_2_) to be reduced from the initial 0.4 to 0.21. Pneumonia resolved during the intervention period and no adverse events due to MI-E were noted. The patient was discharged with a home mechanical ventilator after 77 days in the hospital.

## Discussion

This report intends to demonstrate that modification of applied airway pressure should be considered when MI-E is used. This is especially important for patients with short height, low BMI, and restricted lung volume because they have been reported at higher risk of pneumothorax [7]. This case report illustrates two important clinical points. First, improvement in atelectasis was achieved even though the MI-E pressure setting was lower than the generally recommended pressure. Second, the pressure setting was adjusted by performing physical examination findings which included chest auscultation, visual chest expansion, and observation of secretion movement towards the tracheal tube. They are feasible in any clinical setting.

In general, recommended pressure from the manufacturer guide is ±40 cmH_2_O for both inspiration and expiration [12]. Winck et al. [8] stated that inspiratory pressure +40 cmH_2_O, expiratory pressure -40 cmH_2_O, and in some cases inspiratory pressure +60 cmH_2_O and expiratory pressure -60 cmH_2_O are effective for expectoration from the oral cavity by MI-E through a mask. However, McDonald et al. [13] reported a Duchenne muscular dystrophy patient who complained of pneumothorax after using MI-E by a mouthpiece. In that report, the set pressure was +50 cmH_2_O in inspiration and -47 cmH_2_O in exhalation. When MI-E pressure is adjusted, PCF is considered as an indicator, because PCF of 160 L/min or higher is a prerequisite for extubation and is considered a guideline for effective airway drainage in adults [14]. Therefore, setting pressure has been titrated to achieve greater PCF. Guerin et al. stated artificial airways significantly reduced PCF with MI-E in a bench study, then they concluded to achieve the desired PCF in patients with artificial airways. The set pressure should be increased [14]. This report may lead clinicians to apply even higher pressure settings for patients with artificial airways when monitoring expiratory flow rate alone. Consequently, the risk of complications from pneumothorax may be increased. A report by McDonald did note that although the incidence of pneumothorax might be low, if a pneumothorax does occur, it is associated with serious outcomes in the NMD patient population [13].

In this case, to avoid unnecessary pressure, we used previous pressure as a baseline and added a minimum amount of pressure (±5 cmH_2_O), then evaluated chest auscultation, visual expansion, and secretions movement, then finally, we set at 25 cmH_2_O. In a study conducted by Kuroiwa et al. [15] on tracheal intubation and tracheostomy patients in the ICU, the authors adjusted pressure while checking the disappearance of rhonchi and improvement of SpO_2_. As a result, inspiratory and expiratory pressures were set at +15/-15 cmH_2_O to +40/-40 cmH_2_O, suggesting that the treatment was effective even at relatively lower pressures [15]. This suggests that the pressure required differs depending both on patients’ response, and other individual conditions.

In the present case, the PCF was 140 L/min, this was less than the target PCF for effective airway clearance. However, the patient had undergone a tracheostomy and had a tracheal cannula inserted, so that even if this patient could not self-extract airway secretions, she was suctioned by health care providers or families, up to the tracheal tube. Therefore, in addition to auscultation of lung parenchyma, auscultation and palpation over the tracheal area are necessary.

Although improved atelectasis was observed, we couldn't indicate exactly how soon the atelectasis has been improved. Initially, bilateral atelectasis was observed, and the right lung improved quickly with antibiotics, expectoration and postural drainage. On the other hand, the left side atelectasis was refractory and did not improve as in the right lung. Then, MI-E has been initiated (Figure 2). No treatment was added other than MI-E in this period. Thus, we presumed MI-E was the most important factor for the improvement of left atelectasis.

In the present experience, the inspiratory and expiratory pressures were set the same. However, Volpe et al. reported [16] that in order to aid in the removal of secretions in MI-E pressure setting, a higher expiratory pressure than the inspiratory pressure (e.g., inspiratory pressure +20 cmH_2_O and, expiratory pressure -30 cmH_2_O) facilitated PCF. It is inferred that such a setting can improve atelectasis and may allow a lower inspiratory pressure setting, reducing the risk of pressure-induced lung injury and providing a safer treatment. This is an issue for future research.

## Conclusions

This case report supported the use of lower ventilating pressures, than what manufacturers generally recommended with MI-E, to reduce the risk of barotrauma and yet prevent atelectasis when caring for an ALS patient with small stature, low BMI, and small lung capacities. We believe that improvement can be expected even at lower pressures, performed in conjunction with treatment response observation. Applying this strategy, an individualized approach will reduce the risk of treatment-related lung injury and provide safer treatment.
